# Fetal Growth Restriction Induces Heterogeneous Effects on Vascular Biomechanical and Functional Properties in Guinea Pigs (*Cavia porcellus*)

**DOI:** 10.3389/fphys.2017.00144

**Published:** 2017-03-10

**Authors:** Daniel Cañas, Emilio A. Herrera, Claudio García-Herrera, Diego Celentano, Bernardo J. Krause

**Affiliations:** ^1^Departamento de Ingeniería Mecánica, Facultad de Ingeniería, Universidad de Santiago de ChileSantiago, Chile; ^2^Programa de Fisiopatología, Facultad de Medicina, Instituto de Ciencias Biomédicas, Universidad de ChileSantiago, Chile; ^3^International Center for Andean Studies, Universidad de ChilePutre, Chile; ^4^Departamento de Ingeniería Mecánica y Metalúrgica, Instituto de Ingeniería Biológica y Médica, Pontificia Universidad Católica de ChileSantiago, Chile; ^5^Division of Pediatrics, Departament of Neonatology, Faculty of Medicine, Pontificia Universidad Católica de ChileSantiago, Chile

**Keywords:** biomechanical, fetal growth restriction (FGR), systemic vessels, umbilical arteries, vascular remodeling

## Abstract

**Aim:** Fetal growth restriction (FGR) is associated with a variety of cardiometabolic diseases in adulthood which could involve remodeling processes of the vascular walls that could start in the fetal period. However, there is no consensus whether this remodeling affects in a similar way the whole vascular system. We aimed to determine the effects of FGR on the vasoactive and biomechanical properties of umbilical and systemic vessels in fetal guinea pigs.

**Methods:** FGR was induced by implanting ameroid occluders at mid-gestation in uterine arteries of pregnant guinea pigs, whilst the control group was exposed to simulated surgery. At the term of gestation, systemic arteries (aorta, carotid and femoral) and umbilical vessels were isolated to determine *ex vivo* contractile and biomechanical responses (stretch-stress until rupture) on a wire myograph, as well as opening angle and residual stresses. Histological characteristics in tissue samples were measured by van Gieson staining.

**Results:** Aorta and femoral arteries from FGR showed an increased in biomechanical markers of stiffness (*p* < 0.01), contractile capacity (*p* < 0.05) and relative media thickness (*p* < 0.01), but a reduced internal diameter (*p* < 0.001), compared with controls. There were no differences in the biomechanical properties of carotid and umbilical from control and FGR fetuses, but FGR umbilical arteries had a decreased contractile response to KCl (*p* < 0.05) along with a reduced relative media thickness (*p* < 0.05).

**Conclusion:** Altogether, these changes in functional, mechanical and morphological properties suggest that FGR is associated with a heterogeneous pro-constrictive vascular remodeling affecting mainly the lower body fetal arteries. These effects would be set during a pathologic pregnancy in order to sustain the fetal blood redistribution in the FGR and may persist up to adulthood increasing the risk of a cardiovascular disease.

## Introduction

Fetal growth restriction (FGR) is a condition in neonates whose birth weight is below the 10th percentile for its gestational age, but in a comprehensive manner, represents any condition that constraint or negatively alter fetal growth trajectory (Zhang et al., 2010). FGR babies have at short term an increased perinatal morbidity and mortality, whilst at long term these subjects have an important impairment of their development and a greater risk of developing cardiometabolic diseases at adulthood (Barker, 2006; Hunter et al., 2016). It has been proposed that the cardiovascular risk associated to FGR is a consequence of morphological and functional alterations in the fetal arteries, which would derive from the chronic blood flow redistribution that takes place when nutrient and oxygen delivery to the fetus is restricted (Harman and Baschat, 2003). Few studies have analyzed the biomechanical characteristics of vessels derived from fetuses affected by FGR, suggesting the presence of an increased arterial stiffness resulting from increased collagen content and remodeling of the arterial wall. These changes would impair at short and long term the vascular structure and function (Dodson et al., 2013, 2014). However, none of these studies have addressed the relationship between the biomechanical and vasoactive responses of different arteries derived from FGR subjects.

Compelling data show that FGR babies have a pro-atherogenic vascular structure, characterized by an increased aortic intima-media thickness (Skilton et al., 2005) and stiffness (Dodson et al., 2013), which seem to be permanent across the lifespan. Comparable effects of FGR on aorta remodeling have been reported in experimental models of FGR in sheep and guinea pigs (Thompson et al., 2011; Dodson et al., 2014). However, there is no clarity whether these structural alterations represent common changes in the entire circulatory system or are territory-specific. Moreover, different studies addressing the presence of vascular remodeling in the carotid artery at birth (Crispi et al., 2012; Morsing et al., 2014; Stergiotou et al., 2015), as well as at different ages (Martin et al., 2000; Oren et al., 2004; Te Velde et al., 2004; Bjarnegard et al., 2013), show no conclusive effects of FGR in humans. A single study in carotid arteries from FGR fetal sheep suggests that vascular remodeling at this level would be opposite to the morpho-structural changes observed in the aorta (Dodson et al., 2013, 2014). Nonetheless, there are no studies comparing the impact of FGR on the structure and function of vessels from different vascular beds and how these changes reflect the chronic blood redistribution that occurs in the restricted fetuses.

Classical analysis of vascular alterations in pathological conditions has been addressed using histological or *ex vivo* functional specific approaches, which correlate in some extend with the effects observed *in vivo* (Mulvany and Aalkjaer, 1990). Similarly, analysis of the biomechanical properties of vessels samples exposed to non-physiological forces has been extensively used to unveil structural changes in the vascular tree occurring in adult subjects with cardiovascular dysfunction (Weisbecker et al., 2015). The present study aims to characterize the vascular changes that take place in the FGR vasculature, by comparing histological and biomechanical properties, as well as *ex vivo* functional responses in carotid, aorta, umbilical and femoral arteries from control and FGR fetuses. We hypothesized that FGR has a differential effect on the biomechanical and structural properties of the fetal aorta, carotid, femoral and umbilical arteries and these heterogenous changes reflect the blood flow redistribution occurring in this condition. Using a blind scheme with the three complementary approaches, we aimed to provide a comprehensive modeling of the structural changes in the FGR circulatory system.

## Materials and methods

### Animals

All animal care, measurements, and experimental procedures were approved by the Ethics Committee of the Faculty of Medicine of the Pontificia Universidad Católica de Chile (1130801) and the Universidad de Chile (protocol CBA N° 0694 FMUCH) and were conducted according to the Guide for the Care and Use of Laboratory Animals published by the US National Institutes of Health (NIH Publication No. 85–23, revised 1996). These procedures were reported in accordance with the ARRIVE guidelines (https://www.nc3rs.org.uk/arrive-guidelines).

Fourteen adult female Pirbright White guinea pigs (*Cavia porcellus*) were used for this study. All animals were housed in individual cages under standard conditions (30–35% humidity, 20–21°C and a 12:12-h light-dark cycle), with controlled food-by-body weight intake with a commercial diet (LabDiet 5025, Guinea Pigs, 20–30 g d^−1^) and alfalfa hay, plus water *ad libitum*. Four-to five-month-old virgin sows in estrus were paired with a fertile male for 2 days. After the mating period, the females were individually housed with daily monitoring of body weight, food intake, and water consumption. Pregnancy was confirmed with ultrasonography at d 20–25, where the first day with the male was considered day 0 of pregnancy (Term ~67 days).

### Surgical FGR induction

At day 35 of gestation, all pregnant sows were subjected to aseptic surgery, randomly assigned to either sham-operated (control) or progressive uterine artery occlusion (FGR) as previously described (Herrera et al., 2016). Briefly, under general anesthesia (ketamine 60 mg kg^−1^, xylazine 4 mg kg^−1^ and Atropine 0.1 mg kg^−1^, IM) an infra-umbilical midline laparotomy was performed, exposing the gravid uterus. For the FGR group, ameroid constrictors (COR-2.00-SS, NW Research Instruments, Inc., USA) were placed bilaterally around the base of each uterine artery. The abdominal wall and skin were then sutured in layers with absorbable sutures (Vicryl 4/0, Ethicon, USA). Finally, surgical staples (Auto Suture™, Condivien, Dominican Republic) were installed in the skin. As part of this procedure, animals received analgesia (carprofen 4 mg kg^−1^, SC) and prophylactic antibiotic (20 mg oxytetracycline kg^−1^, SC) treatments. The skin staples were removed 7–8 days after surgery. The control group underwent the same surgical, analgesic and prophylactic procedures, but without placement of the ameroid constrictors (sham-operated).

### Euthanasia at near-term

At ~90% of pregnancy, approximately 60–63 days of gestation, the guinea pigs, and their fetuses were euthanized with a maternal anesthetic overdose (Sodium Thiopentone 200 mg kg^−1^, IP, Opet, Laboratorio Chile). Once the cardio-respiratory arrest was confirmed, the fetuses were extracted and their umbilical, aorta, carotid and femoral vessels were carefully dissected.

### Passive response

Vessel segments of 2 mm of carotid, aorta, femoral and umbilical arteries were mounted in a wire myograph (model 620M; Danish Myo Technology A/S, Aarhus, Denmark), maintained at 37°C in Ca^2+^-free Krebs buffer (in mmol L^−1^: 118.5 NaCl, 25 NaHCO_3_, 4.7 KCl, 1.2 KH_2_PO_4_, 1.2 MgSO_4_, 5.5 D-glucose) with constant bubbling (5% CO_2_ in air).

The passive response measurement consists in subjecting to vessel segment to a radial elongation performed by hooks. During the test, the load (*F*) and the displacement of the hooks (Δ) were recorded (Bustos et al., 2016). The variables used were the initial thickness (*e*_*o*_), the initial width (*a*_*o*_), the diameter of the hook (ϕ) and the mean diameter of the artery (*d*). The initial length (Δ_*o*_) was determined by:

(2.1) $$\Delta_{o}=\frac{\pi }{2}\left[d-\left(\phi +e_{o}\right)\right]$$

Thus, it is possible to define the elongation considering the semi-perimeter of the artery and the increase in separation of the hooks:

(2.2) $$\lambda =1+2\frac{\left(\Delta -\Delta_{o}\right)}{\pi d}$$

Finally, the expression of the Cauchy stress in the arterial wall was defined by:

(2.3) $$\sigma =\frac{F}{2a_{o}e_{o}}\lambda$$

**Figure 1:**
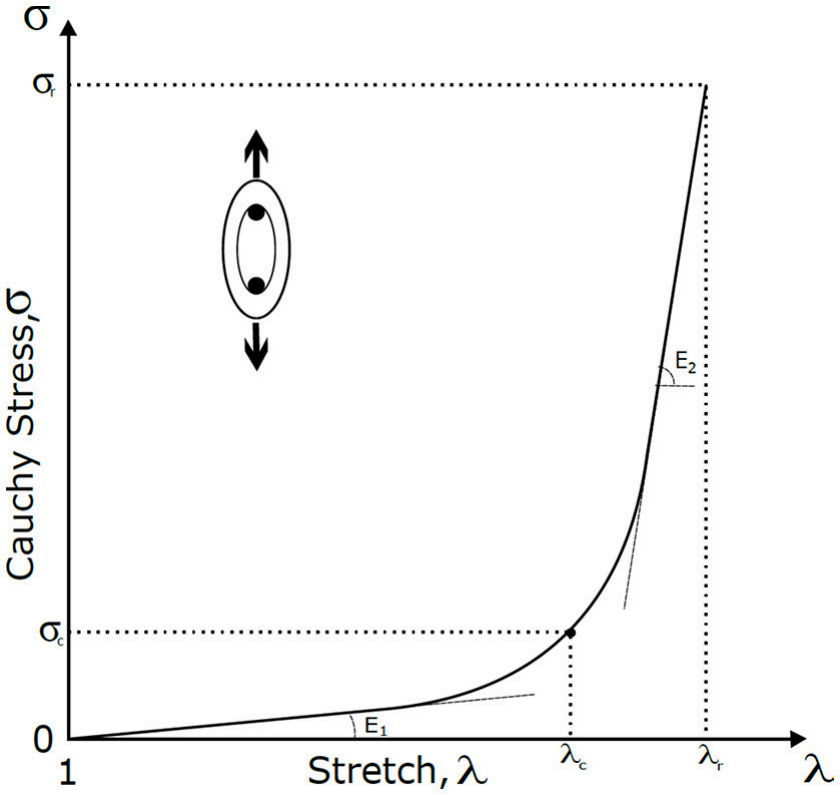
**Stress-stretch curve and mechanical parameters**. Schematic representation of the parameters displayed in experimental stress-stretch curves; the stretch (λ, an index of deformation of the material), the Cauchy stress (σ, measure of the internal forces per unit of area), the rupture stretch (λ_r_, represent the stretch at the breaking point), the rupture stress (σ_r_, represent the stress at the breaking point), the elbow stress (λ_c_, transition point between the elastic and stiff zone), the initial slope (E_1_, represents the contribution of the elastin to the stiffness) and the final slope (E_2_, associated mainly with the collagen fibers).

To simplify the analysis and allow the comparison between specimens, five parameters were summarize to represent the passive mechanical response of the arterial wall, used in previous works (Garcia-Herrera et al., 2012); the magnitude of the slope at the beginning (E_1_) and at the end (E_2_) of the stress-stretch curve, the stress at the elbow of the stress-stretch curve (σ_c_) and the stretch and stress at the breaking point (λ_R_, σ_R_) (Figure 1).

### Active response

Vessel segments of 2 mm of carotid, aorta, femoral and umbilical arteries were dissected and mounted in a wire myograph (model 620M; Danish Myo Technology A/S, Aarhus, Denmark), maintained at 37°C in Krebs buffer with constant bubbling (5% CO_2_ in air, a gas mixture that delivered ~100 mmHg of oxygen in the buffer) (Wareing et al., 2006). Isometric force was recorded using a PowerLab data acquisition hardware (ADInstruments, Castle Hill, Australia) and LabChart software (version 6; ADInstruments). After 30 min of equilibration, vessel internal circumferences were determined by measuring the maximal active force in response to KCl (65 mmol L^−1^) as described (Krause et al., 2016). This method allows the comparison between different vessels normalizing the vessel tone to similar *in vivo* levels (Mulvany and Aalkjaer, 1990). Maximal wall tension was determined by measuring the tension achieved to increasing concentration of KCl (5–125 mmol L^−1^) and the vessel length as previously described (Delaey et al., 2002).

### Ring opening test

For the determination of residual stress in the different arteries studied, opening angle measurements in vessels rings were performed. The opening angle (Garcia-Herrera et al., 2016) is used to measure the angle (α) subtended by the ends after making a radial cut of a circular segment of the artery. Briefly, the ring was immersed in calcium-free Krebs at 37°C ± 1°C for about 5 min, and subsequently cut radially and photographed after 20 min. With this, the internal diameter is obtained from the circumference that best fit the open ring and the mean thickness was considered as the mean of 5 measurements of each sample.

### Vessel histology

After dissection, freshly isolated vessels segments of fetal carotid, aorta, femoral and umbilical arteries were immersed in 4% formaldehyde for 24 h and then washed in PBS 1 × and embedded in paraffin. Thereafter, sections were cut in 5 μm serial slides and treated with Hematoxylin-eosin and van Gieson staining procedures (Herrera et al., 2016). Histological sections of fetal carotid, aorta, femoral and umbilical arteries were analyzed and photographed at 10 × or 40 × with a microscope (Olympus BX-41) coupled to a digital camera. Briefly, luminal, medial and adventitial perimeters were measured for the estimation of the internal and, external diameters. Further, luminal, medial and adventitial areas were measured and the following ratios calculated: luminal/vascular area and luminal/wall area as indexes of vascular remodeling (Herrera et al., 2008). All measurements were performed in 5 replicates and an average per animal was calculated. The analysis of the microphotographs was performed with the software Image Pro-Plus.

### Statistical analysis

Values are expressed as mean ± S.E.M., where n indicates the number of animals per analyses. The different approaches were carried out in independent laboratories with a blind scheme to avoid bias. Data from isolated vessels reactivity were adjusted to Boltzmann sigmoidal curves from which maximal responses and potency (EC_50_) were obtained. All comparisons were analyzed by ANOVA One way. Analyses were carried out with GraphPad Prism 6.01 (GraphPad Software Inc., San Diego, CA, USA), where *p* ≤ 0.05 was considered the cut-off for statistical significance.

## Results

### Biomechanical properties and residual stress in systemic and umbilical arteries from FGR fetuses

**Figure 2:**
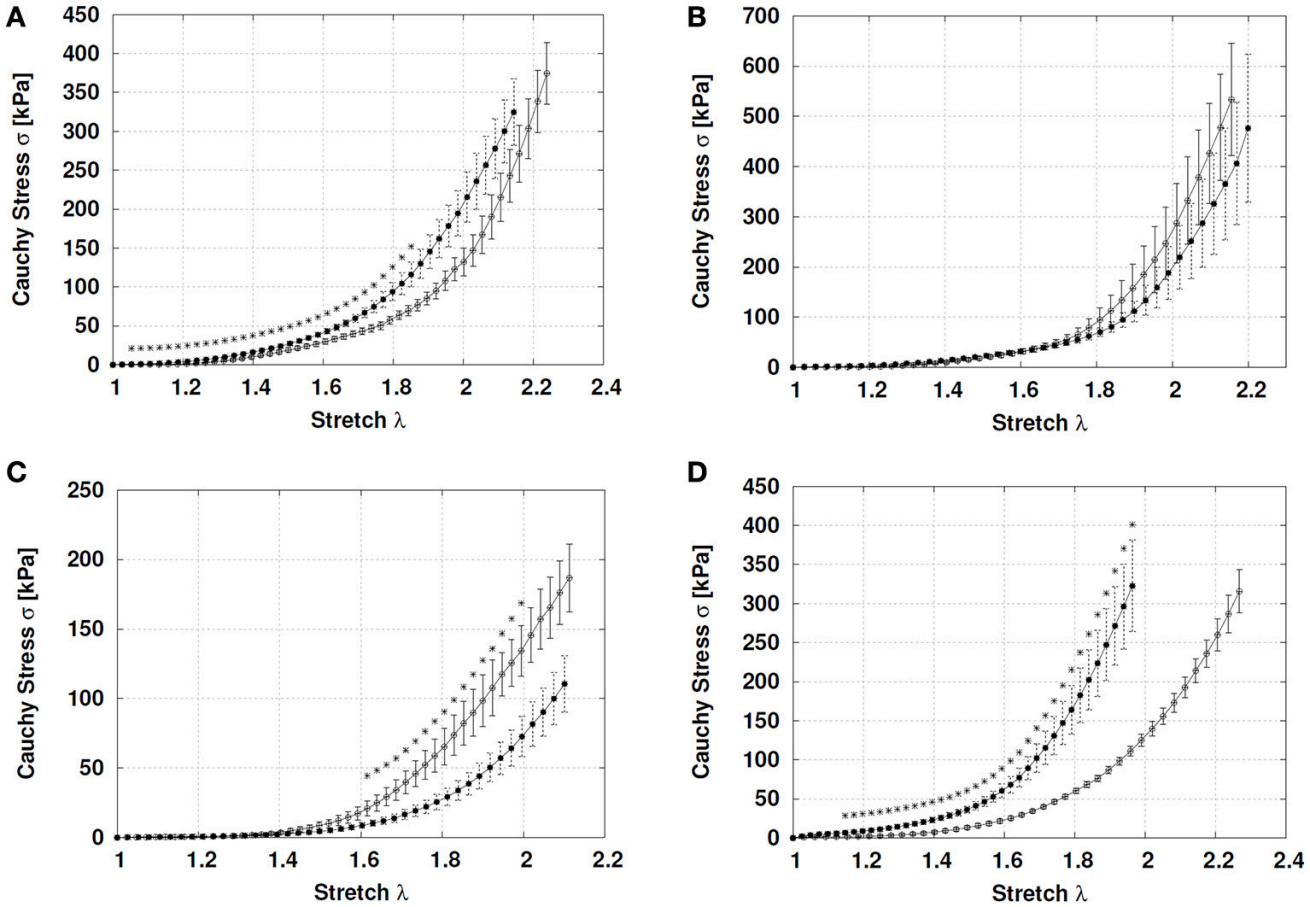
**Stress-stretch curves in systemic and umbilical arteries from guinea pig fetuses**. Stress-stretch for **(A)** aorta, **(B)** common carotid, **(C)** umbilical, and **(D)** femoral arteries from control (open circles, *n* = 7 different subjects) and FGR (solid circles, *n* = 6 different subjects) fetal guinea pigs until the first sample rupture. Values were expressed as Mean ± SEM. ^*^p < 0.05, *t*-test.

In FGR aorta there was an increase in the initial slope (15.490 ± 1.349 kPa) compared to control (5.246 ± 1.895 kPa) (*p* = 0.003), but similar final slopes (control, 1041 ± 114 kPa; FGR, 1363 ± 152 kPa, *p* = 0.10). The elbow of the experimental curve was defined by the point [λ, 2.026; 146.15 kPa] for control and [λ, 1.820; 102.46 kPa] for FGR, without differences in the rupture points (λ_control_ = 2.318, 527.6 ± 72.2 kPa; λ_FGR_ = 2.321, 512.6 ± 80.7 kPa, *p* = 0.89) (Figure 2A).

Carotid arteries from control and FGR showed comparable initial (control, 6.43 ± 1.87 kPa; FGR, 10.590 ± 3.924 kPa, *p* = 0.32) and final (control, 2178 ± 293 kPa; FGR, 1749 ± 288 kPa, *p* = 0.32) slopes. Similarly, the elbow of the experimental curve ([λ_control_, 1.916; 176.39 kPa] [λ_FGR_, 1.923; 129.13 kPa]) and rupture points (λ_control_ = 2.406; 1023 ± 117 kPa; λ_FGR_ = 2.499; 898 ± 190 kPa) were comparable between control and FGR carotid arteries (Figure 2B).

There were no differences in the initial (control, 4.289 ± 1.065 kPa; FGR, 4.012 ± 1.013 kPa, *p* = 0.85) and final (control, 502.3 ± 40.9 kPa; FGR, 548.2 ± 57.86 kPa, *p* = 0.54) slopes between control and FGR umbilical arteries (Figure 2C). However, point to point comparison showed a lower Cauchy stress at high stretch conditions (*p* < 0.05) in FGR arteries. The elbow of the experimental curve was defined by the point [λ, 1.716; 41.50 kPa] for control and [λ, 1.868; 39.54 kPa] for FGR, whilst rupture points were similar between control (λ = 2.184, 225.3 ± 10.33 kPa) and FGR (λ = 2.31, 206.1 ± 24.64 kPa) umbilical arteries.

Contrariwise, FGR femoral arteries showed a higher initial slope (32.19 ± 5.31 kPa) compared with control (6.43 ± 1.87 kPa) (*p* = 0.002) without differences in the final slope (control, 1217 ± 123 kPa; FGR, 1439 ± 159 kPa, *p* = 0.34). The elbow of the experimental curve was defined by the point [λ, 1.966; 114.80 kPa] for control and [λ, 1.699; 106.07 kPa] for FGR. Rupture points were comparable between control (λ = 2.552; 652 ± 85 kPa) and FGR (λ = 2.305; 638 ± 59 kPa) femoral arteries (*p* = 0.90) (Figure 2D).

In the opening ring test, there were an increase in the opening angle in the FGR aorta (+55.63%) and carotid (+17.65%) arteries, but a decrease in umbilical (−16.40%) and femoral (−25.16%) arteries compared with the control group (Table 1).

**Table 1:** **Opening angle measurements for arteries from control and FGR fetuses**.

		**Control**	**FGR**	***p***
Carotid	(α, °)	86.10 ± 4.01	101.30 ± 6.86	0.0567
Aorta	(α, °)	55.74 ± 3.03	86.75 ± 4.16	**0.0003**
Umbilical	(α, °)	86.02 ± 5.26	71.91 ± 3.25	**0.0497**
Femoral	(α, °)	135.10 ± 4.72	101.10 ± 3.57	**0.0001**

*Values expressed as Mean ± SEM, t-test. Results with significant statistical differences are indicated in bold*.

### *Ex vivo* constrictor responses in systemic and umbilical arteries from FGR fetuses

**Figure 3:**
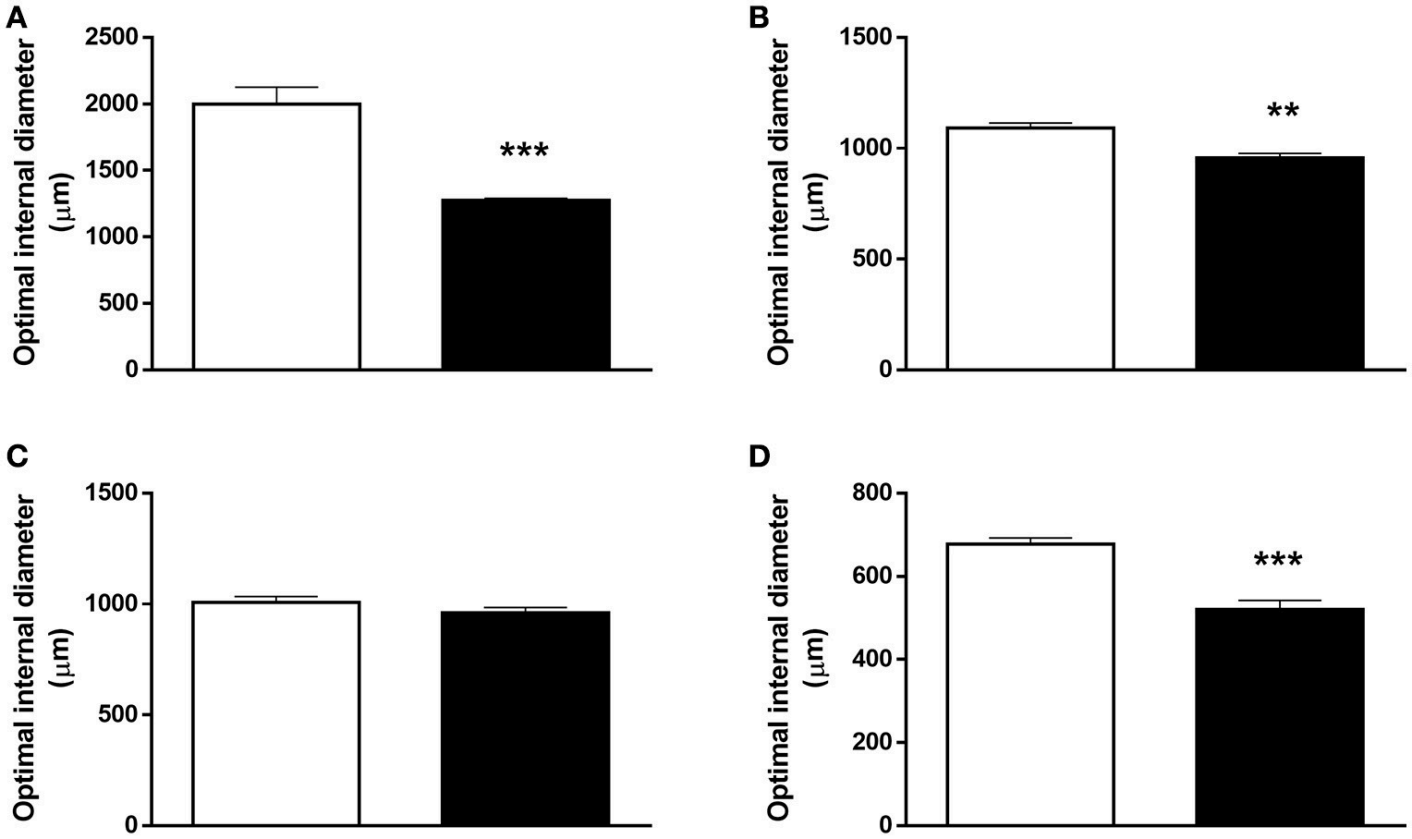
**Optimal diameter in systemic and umbilical arteries from guinea pig fetuses**. Optimal diameter determined by measuring the maximal active force in response to KCl in **(A)** aorta, **(B)** common carotid, **(C)** umbilical, and **(D)** femoral arteries from control (open bars, *n* = 7 different subjects) and FGR (solid bars, *n* = 6 different subjects) fetal guinea pigs. Values were expressed as Mean ± SEM. ^**^p < 0.01, ^***^p < 0.001 *t*-test.

Aorta, carotid and femoral arteries (Figures 3A,B,D) from FGR showed a decrease of 13, 35, and 25% in the optimal diameter, respectively, compared with their control counterparts, whilst no differences in the optimal diameter were observed in umbilical arteries (Figure 3C).

**Figure 4:**
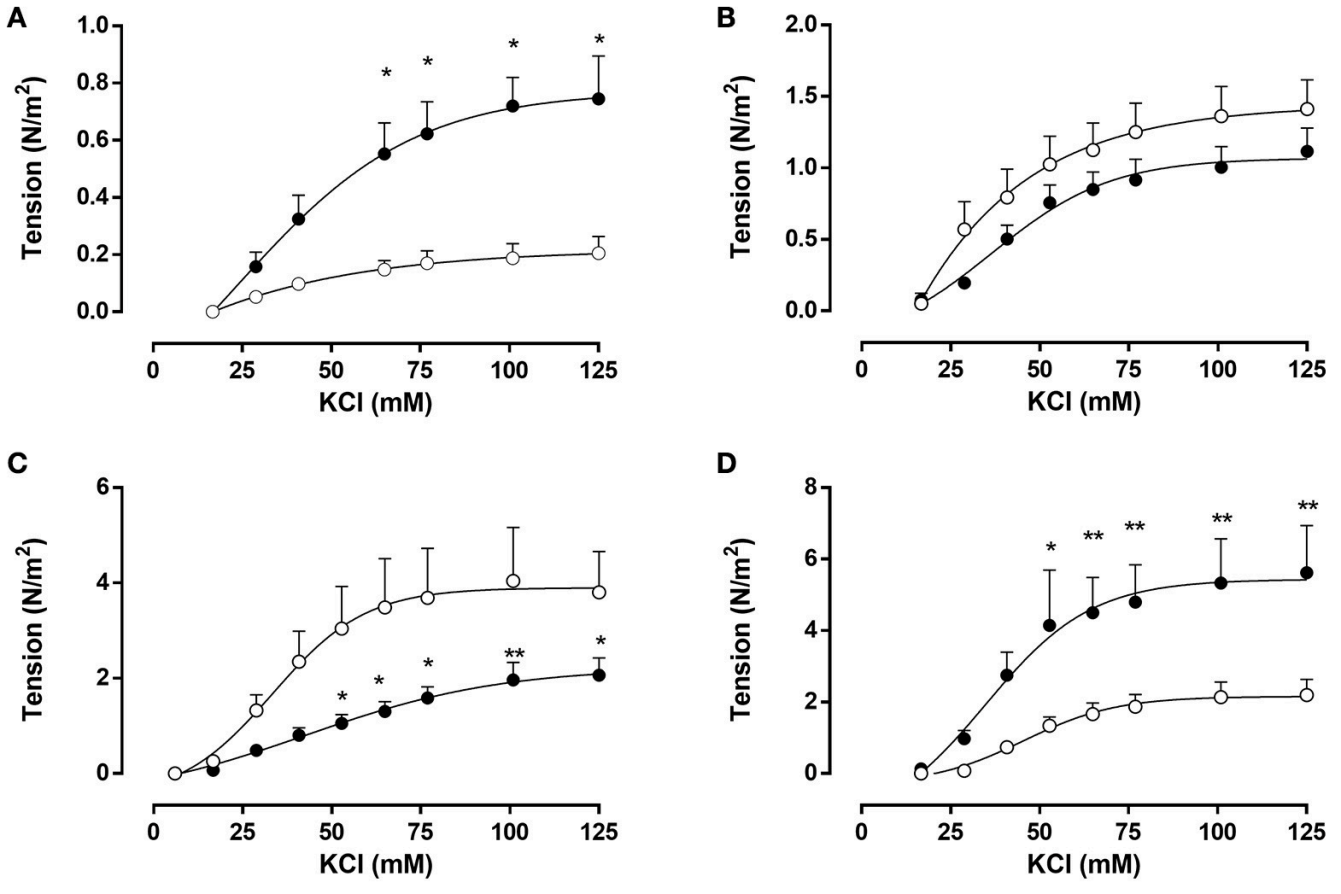
***Ex vivo* contractile response to KCl in systemic and umbilical arteries from guinea pig fetuses**. Concentration-response curves in response to KCl from **(A)** aorta, **(B)** common carotid, **(C)** umbilical, and **(D)** femoral arteries from control (open bars, n = 7 different subjects) and FGR (solid bars, n = 6 different subjects) fetal guinea pigs. Values were expressed as Mean ± SEM. ^*^p < 0.05, ^**^p < 0.01 vs. control, ANOVA.

Conversely, FGR aorta showed a 3.7-fold increase in the maximal tension in response to KCl (0.8 ± 0.1 N m^−2^) compared with controls (0.2 ± 0.1 N m^−2^) (Figure 4A), an effect also observed in FGR femoral arteries (Figure 4D) (FGR, 5.4 ± 0.7 N m^−2^ vs. controls, 2.2 ± 0.2 N m^−2^). The increased response to KCl in FGR aorta and femoral arteries were evident from concentrations of KCl above 50 mM. In contrast, vasoactive constriction in response to KCl was comparable between control and FGR in carotid arteries with a maximal tension of ~1.2 N m^−2^ (Figure 4B). Furthermore, FGR umbilical arteries (Figure 4C) showed a lower KCl-induced constriction relative to controls (FGR, 2.2 ± 0.4 N m^−2^ vs. controls, 3.9 ± 0.5 N m^−2^), a difference observed at elevated KCl concentrations (>50 mmol L^−1^).

### Histology of systemic and umbilical arteries from FGR fetuses

Morphometry of vessel samples showed a decreased area of the intima (~30%) and adventitia (~35%) but an increased media (~15%) in FGR aorta relative to control arteries. Vascular dimensions were similar for the carotid arteries from control and FGR fetuses. In contrast, there was a decrease in the media (~15%) and an increase in the area of the adventitia (~35%) compared with control samples in FGR umbilical arteries. Conversely, FGR femoral arteries showed a decreased in the intima (~30%) and adventitia (~25%) but an increase in the media (~30%) (Table 2).

**Table 2:** **Histological properties of systemic and umbilical arteries**.

		**Control**	**FGR**	***p***
Carotid	Intima (%)	7.42 ± 0.25	8.00 ± 0.25	0.1619
	Media (%)	56.07 ± 1.70	59.67 ± 1.02	0.1195
	Adventitia (%)	36.51 ± 1.87	32.33 ± 1.15	0.1061
Aorta	Intima (%)	7.48 ± 0.31	5.26 ± 0.41	**0.0210**
	Media (%)	72.52 ± 0.37	82.00 ± 0.27	**0.0004**
	Adventitia (%)	20.00 ± 0.50	12.74 ± 0.68	**0.0053**
Umbilical	Intima (%)	2.01 ± 0.34	1.43 ± 0.16	0.1613
	Media (%)	72.31 ± 2.24	63.74 ± 1.97	**0.0207**
	Adventitia (%)	25.68 ± 1.94	34.82 ± 1.22	**0.0040**
Femoral	Intima (%)	11.92 ± 0.42	7.99 ± 0.83	**0.0063**
	Media (%)	47.72 ± 3.61	62.92 ± 2.47	**0.0089**
	Adventitia (%)	40.36 ± 3.47	29.09 ± 1.75	**0.0174**

*Values expressed as Mean ± SEM, t-test. Results with significant statistical differences are indicated in bold*.

## Discussion

In this work, we studied and compared the changes in the mechanical, functional and structural properties of the aorta, carotid, umbilical and femoral arteries from guinea pig fetuses affected by fetal growth restriction. The biomechanical test showed a normal response to stretch in carotid and umbilical arteries from FGR, however, there was an increased stiffness in the aorta and femoral arteries characterized mainly by higher initial Cauchy stress slope. These biomechanical properties were associated with a normal morphology and response to KCl in carotid arteries, as well as a decreased in the relative media thickness and contractile response to KCl in FGR umbilical arteries. In contrast, the increased biomechanical stiffness in the aorta and femoral arteries from FGR correlated with an increase in the relative intima-media area and KCl-induced constriction. These comparable changes in mechanical, *ex vivo* and morphological properties of the studied arteries suggest that FGR is associated with a heterogeneous vascular remodeling affecting mainly to the lower body fetal arteries.

### Effect of FGR on aorta and femoral arteries

Several studies have addressed the short and long term effects of FGR on vascular function and structure, supporting the notion that an impaired intrauterine growth leads to increasing cardiovascular risk later in life. A study on young adults shows that subjects born with FGR have a decreased internal diameter and increased stiffness in the ascending aorta (Bjarnegard et al., 2013). In this context, data from hypoxia-induced FGR in guinea pigs demonstrate the presence of vascular remodeling in the aorta of adult animals (Thompson et al., 2011). Similar changes in the *in vivo* structural and biomechanical properties of the aorta have been reported in children born with FGR (Bradley et al., 2010; Zanardo et al., 2011) suggesting that this altered vascular structure has an early onset. In this study, using a guinea pig model of FGR, we found that the aorta and femoral arteries from FGR subjects have a stiffened response to stress-stretch, along with an increased contractile capacity and reduced internal diameter which were associated to remodeling traits. A comparable study in fetal sheep show similar changes in the biomechanical properties of the aorta from FGR, however, those changes were not paralleled by modifications in the vessel morphology and no data regarding the vasocontractile response is provided (Dodson et al., 2014). Compelling evidence in humans show that FGR is associated with an increased aortic intima-media thickness and decreased internal diameter (Skilton et al., 2005; Koklu et al., 2007; Visentin et al., 2013; Zanardo et al., 2013; Stergiotou et al., 2015) which can be evidenced even *in utero* (Cosmi et al., 2009; Gomez-Roig et al., 2015). Interestingly, the increase in aortic intima-media thickness in human FGR is associated with an increase in the umbilical artery pulsatility index (Cruz-Lemini et al., 2014) an effect also observed in this model (Herrera et al., 2016). It is worth to note that, changes in biomechanical properties of FGR aorta and femoral arteries in this study were not completely comparable. In FGR aorta the most significant changes occurred in the maximal contractile response (4-fold higher than control) and residual stress (50% increase), whilst in femoral arteries there was a reduction in the residual stress but a substantial increase in the Cauchy stress slope (5-fold higher than control) which could be due to the different nature of these arteries. Altogether, this data suggests that FGR is associated with an early pro-constrictive vascular remodeling, affecting conduit and peripheral arteries which could contribute to cardiovascular diseases later in life.

### Effect of FGR on carotid and umbilical arteries

Considering that carotid artery intima-media thickness has been extensively used as a prognostic tool for cardiovascular disease, several studies have attempted to determine a relationship between born with FGR and structural alterations in the carotid artery. Notably, studies in adults show no changes in the intima-media thickness and biomechanical properties in the common carotid artery from subjects born FGR (Oren et al., 2004; Te Velde et al., 2004; Bjarnegard et al., 2013) or with a reduced birth weight (Painter et al., 2007). Furthermore, studies in children and neonates born with FGR show no conclusive data regarding modifications in the carotid artery structure or biomechanical properties (Martin et al., 2000; Crispi et al., 2012; Morsing et al., 2014; Stergiotou et al., 2015). In this study, we found a decrease in the internal diameter of FGR carotid arteries, however, there were no changes in the biomechanical, contractile and morphological properties of these arteries compared with control. Conversely, it has been reported in growth-restricted fetal sheep a decrease in the carotid artery intima-media thickness and increased internal diameter and Cauchy stress (Dodson et al., 2013). In spite of the differences in both studies, it has been extensively reported that FGR is associated with a preserved, or even favored, blood flow to the brain (Giussani, 2016), which would be accompanied by permissive biomechanical properties in carotid arteries.

Interestingly, FGR umbilical arteries show counterintuitive changes in their biomechanical and morphology properties. It is well established that, in humans, an increased placental vascular resistance occurs in FGR which is commonly expressed as an increased umbilical artery pulsatility and resistance index. Nonetheless, several studies show that this increased vascular tone is associated with a reduction in the umbilical artery wall thickness (Bruch et al., 1997; Yoshimatsu et al., 2006; Burkhardt et al., 2009; Sharony et al., 2016) and maximal contractile force in response to KCl (Krause et al., 2013). Therefore, the increase in umbilical artery pulsatility and resistance index mainly represents downstream placental vascular resistance, which correlates with FGR and multisystem effects of placental deficiency (Harman and Baschat, 2003). In the present study, there were no significant differences in the Cauchy stress curves between control and FGR guinea pig umbilical arteries; however, there was a substantial decrease in the residual stress and intima-media area which translated into a decreased maximal contractile response. These changes in the umbilical artery could result from an increased proliferation but a reduce the size of smooth muscle cells (Yoshimatsu et al., 2006) as well as lower relative content and less organized elastin (Dodson et al., 2013).

### Integrating the vascular remodeling in the FGR

It is widely accepted that asymmetric FGR is accompanied by a blood flow redistribution toward the upper body in order to maintain brain nutrient and oxygen supply, a term named brain sparing (Giussani, 2016). It has been proposed that this redistribution would occur accompanied by a conserved, or even permissive, vascular function in arteries that feed cerebral circulation, such as carotid arteries, and an increased resistance in peripheral arteries from the lower body, such as femoral arteries. However, *in vivo* studies in animal models of impaired fetal growth conditions show heterogeneous effects on blood flow redistribution to the lower body (Poudel et al., 2015; Allison et al., 2016) suggesting the need for further evidence. In this study, using a guinea pig model of asymmetric FGR we found a differential effect of an impaired placental function on the biomechanical and morphology properties of systemic fetal arteries feeding the upper and lower body. However, permissive remodeling was also observed in umbilical arteries (a lower body vascular bed), suggesting that a vascular remodeling favoring prefusion would occur in other organs that show a preserved growth in FGR. It is worth to note that the characterization made in this report gives a reasonable approximation of the pathological mechanical and vasoactive behavior of these vascular beds, but we cannot quantify the functional consequences. Nonetheless, we recently reported that in this animal model there is a reduced vasodilatory response to acetylcholine, a sign of endothelial dysfunction in aorta, femoral and umbilical arteries (Herrera et al., 2017), such as have been reported elsewhere. Future studies must take into account the active response considering changes in the vascular tone, in addition to detailed analysis of the microstructure, providing phenotyping of the different cell types. Nonetheless, altogether this data suggests that FGR leads to a differential artery remodeling which would privilege cerebral and placental circulation and established a pro-constrictive biomechanical and vasoactive behavior in the peripheral circulation.

## Author contributions

EH, CG, DC, and BK conceived and designed the experiments. DC, EH, and BK collected, analyzed and interpreted the experimental data. DC, EH, CG, DCe, and BK drafted the article, and all authors revised it critically and approved the final version.

### Conflict of interest statement

The authors declare that the research was conducted in the absence of any commercial or financial relationships that could be construed as a potential conflict of interest.
